# A Phase I/II Study of Ultra-Hypofractionated Carbon-ion Radiation therapy for Low- and Intermediate-Risk Localized Prostate Cancer

**DOI:** 10.1016/j.adro.2024.101705

**Published:** 2025-01-11

**Authors:** Noriyuki Okonogi, Hiroshi Tsuji, Kana Kobayashi, Mio Nakajima, Shuri Aoki, Takanobu Utsumi, Hiroyoshi Suzuki, Koichiro Akakura, Tomohiko Ichikawa, Hitoshi Ishikawa

**Affiliations:** a QST Hospital, National Institute for Quantum Science and Technology, 4-9-1 Anagawa, Inage-ku, Chiba City, Chiba, 263-8555, Japan; b Department of Radiation Oncology, Juntendo University Graduate School of Medicine, 2-1-1 Hongo, Bunkyo-ku, Tokyo 113-8421, Japan; c Department of Urology, Toho University Sakura Medical Center, 564-1 Shimoshizu, Sakura-shi, Chiba 285-8741, Japan; d Department of Urology, Japan Community Health Care Organization Mishima General Hospital, 2276 Fujikubo, Yata, Mishima, Shizuoka 411-0801, Japan; e Department of Urology, Graduate School of Medicine, Chiba University, 1-8-1 Inohana, Chuo-ku, Chiba-shi, Chiba 260-8670, Japan

## Abstract

**Purpose:**

We report herein the 3-year results of a phase I/II prospective study of 4-fraction course of carbon-ion radiation therapy (CIRT) in patients with localized prostate cancer.

**Methods and Materials:**

The present was a single-institution, phase I/II prospective study including patients with low- or intermediate-risk prostate cancer, as defined by the National Comprehensive Cancer Network criteria. Eligible patients were randomly assigned (1:1) to a 1- or 2-week schedule. Dose-limiting toxicities (DLTs) were defined as any genitourinary (GU) or gastrointestinal (GI) toxicity grade 3 or higher within 90 days of beginning CIRT. Ten patients were enrolled in each group, and the CIRT dose was increased in a stepwise manner if there were fewer than 4 cases of DLT. The initial CIRT dose was 36 Gy, followed by 40 Gy or 44 Gy. Low-risk patients did not receive androgen deprivation therapy (ADT), whereas intermediate-risk patients received 4 to 8 months of neoadjuvant ADT.

**Results:**

Between October 2018 and October 2020, 60 patients were enrolled in the present study and completed the treatment regimen. The median post-CIRT follow-up period was 42 months (range, 27-59 months). Of the 60 patients enrolled, 10 were in the low-risk group, and 50 were in the intermediate-risk group. Neither group experienced grade 3 or higher GI or GU adverse events; therefore, no dose-limiting toxicities were observed. The incidence of grade 2 GU toxicity within 90 days post CIRT was significantly higher in the 44 Gy group than in the 36 to 40 Gy group (*P* < .01, chi-square test with Yates correction). Biochemical failure was observed in 3 cases by 3 years post CIRT. No clinical recurrence or death because of prostate cancer occurred.

**Conclusions:**

Forty Gy in 4 fractions of CIRT may be appropriate for balancing the therapeutic effects and toxicity. Our findings support further investigations into the efficacy of this strategy.

## Introduction

Prostate cancer is one of the most common cancers among men worldwide, with >1.4 million new cases of prostate cancer reported in 2020.^1^ Prostate cancer is the most common cancer in men in the United States, affecting 1 of every 8 men sometime in their lifetime.^2^ Most patients are diagnosed with potentially curable prostate cancer through prostate-specific antigen (PSA) screening^3^; therefore, the majority of patients with prostate cancer are diagnosed with an indication for local treatment, such as surgery or radiation therapy (RT).

RT schedules for prostate cancer have been getting shorter over the last decade, as several phase III trials have demonstrated that hypofractionated RT, such as using 60 Gy in 20 fractions, was not inferior to conventional fractionation using 74 Gy in 37 fractions or 78 Gy in 39 fractions.^4^^,^^5^ Data on patient-reported quality of life also showed no difference among patient-reported outcomes at 5 years between standard and moderately hypofractionated regimens.^6^ The American Society for Radiation Oncology, in collaboration with the American Society of Clinical Oncology and the American Urological Association, has stated that the accumulated data were sufficiently robust to justify the routine use of moderate hypofractionation in clinical practice for the treatment of localized prostate cancer.^7^ Furthermore, recent advancements in RT technology have produced promising results for ultrahypofractionated RT of 5 to 7 fractions in patients with localized prostate cancer.^8^, ^9^, ^10^ The implementation of shorter RT schedules in patients with prostate cancer, therefore, is now a significant and noteworthy trend.

Carbon-ion RT (CIRT) has distinct biologic and physical properties compared to proton or photon RT: a higher cell-killing potential because of higher linear energy transfer, as well as superior dose distribution as a result of the Bragg peak and minimal lateral scattering.^11^^,^^12^ Clinical trials of CIRT for patients with prostate cancer have been conducted separately from photon RT because of the different properties of CIRT versus photon RT.^13^, ^14^, ^15^, ^16^, ^17^ A recent multi-institutional prospective study of 2157 patients treated with CIRT for prostate cancer demonstrated excellent clinical outcomes.^17^ The 5-year biochemical relapse-free survival (bRFS) rates in the low-, intermediate-, and high-risk groups were 92%, 89%, and 92%, respectively. The results of late adverse events (AEs) were also favorable, and the incidences of grade 2 genitourinary (GU) and gastrointestinal (GI) toxicities were 4.6% and 0.4%, respectively, whereas there were no grade 3 or higher toxicities.^17^ Based on these favorable outcomes, CIRT for patients with localized or locally advanced prostate cancer has been covered by the National Health Insurance of Japan since April 2018.^18^ The current standard schedule of CIRT for prostate cancer is 12 fractions in 3 weeks.^19^ Given the superior dose distribution of CIRT, we hypothesized that further short-term CIRT, namely, ultrahypofractionated CIRT, would be possible for patients with localized prostate cancer. We report, herein, the results of a phase I/II prospective study on ultrahypofractionated CIRT for patients with low- and intermediate-risk localized prostate cancers.

## Methods and Materials

### Study setting and eligibility criteria

The present was a single-institution, parallel-group, phase I/II prospective study conducted at QST hospital. The objectives of the present study were to evaluate the safety and efficacy of CIRT administered in 4 fractions in patients with low- or intermediate-risk prostate cancer, and to determine the appropriate total dose and treatment schedule (1- or 2-week schedule) for these patients.

For the present study, we recruited patients with prostate cancer who intended to undergo radical CIRT as their primary treatment. The inclusion criteria were as follows: (1) biopsy-proven prostate cancer; (2) National Comprehensive Cancer Network low- or intermediate-risk disease; (3) performance status 0-1; (4) expected prognosis of >6 months; and (5) provided written informed consent prior to enrolling. Patients classified as low risk had to meet all of the following criteria: a Gleason score of ≤6; PSA concentration of <10 ng/mL; and clinical or magnetic resonance imaging staging T1c to T2a, N0, and M0 (per the TNM Classification of Malignant Tumors eighth edition). Patients at intermediate risk had at least 1 of the following: clinical stage 2b or T2c; a Gleason score of 7; and/or a PSA concentration of 10 to 20 ng/mL. All patients underwent magnetic resonance imaging prior to their prostate biopsy, and computed tomography and bone scintigraphy prior to enrolling in the present study. The exclusion criteria were as follows: (1) patient underwent therapies other than hormonal therapy for prostate cancer; (2) previous RT to the pelvis; (3) castration-resistant prostate cancer; (4) previous history of transurethral resection of the prostate; (5) active double cancer; or (6) severe pelvic infection. Patients who did not meet the dose constraints for organs at risk were also excluded. The specific dose constraints for the organs at risk are summarized in the ***Treatment*** section below.

Patients were recruited at our hospital, and provided written informed consent prior to enrolling in the present study, the protocol for which was approved by our institutional review board (no. QST: 17-108). Before the start of the study, the study protocol was registered and published in the University Hospital Medical Information Network Clinical Trials Registry (UMIN000032340).

### Patient allocation and study outline

Eligible patients were randomly assigned (1:1) to either a 1- or 2-week schedule. For the 1-week schedule, CIRT was provided for 4 consecutive days, whereas for the 2-week schedule, CIRT was administered twice a week, 4 times in total, and never on consecutive days. The initial dose was prescribed as 36 Gy in 4 fractions, which was escalated to 40 Gy in 4 fractions if dose-limiting toxicities (DLTs) were observed in < patients. Similarly, the dose was escalated to 44 Gy in 4 fractions if DLTs were observed in < 4 patients. Doses were not escalated if 4 or more DLTs were observed in a group. In total, a maximum of 60 patients were planned for enrollment in the present study, with 2 schedules at 3 dose settings, and 10 patients in each group. CIRT doses are conventionally expressed as a photon equivalent dose, “Gy relative biologic effectiveness (RBE),” and defined as the physical dose multiplied by the RBE of CIRT.^20^ In the present study, however, the RBE-weighted doses in CIRT were expressed in Gy, in accordance with the International Commission on Radiation Units and Measurements Report 93 guidelines.^21^

### Endpoints

The primary endpoint of the present study was the incidence of AEs, including DLTs, which were defined DLTs as a GU or GI toxicity grade 3 or higher, based on Common Terminology Criteria for Adverse Events v4.0.^22^ The evaluation period for DLTs was the 90 days following the start of CIRT. Treatment-related AEs 91 days after the start of CIRT were evaluated based on the Radiation Therapy Oncology Group and European Organization for Research and Treatment of Cancer criteria, although they were not defined as DLTs.^23^ The secondary endpoints of the present study were the 3-year bRFS, overall survival (OS), clinical relapse-free survival (CRFS), and disease-specific relapse-free survival (DRFS) rates, which were all calculated from the start date of CIRT. Biochemical failure was defined as an increase >2.0 ng/mL above the nadir PSA,^24^ and if evident, imaging studies were performed to determine whether clinical recurrence was present. The presence of biochemical failure in the absence of clinical recurrence was defined as “clinically non-relapse-free.”

### Treatment

CIRT using pencil beam scanning method was performed at the Heavy-Ion Medical Accelerator in Chiba (National Institute of Radiological Sciences, Chiba, Japan).^25^ The modified microdosimetric kinetic model was applied for RBE-weighted dose calculations.^26^^,^^27^

Clinical target volumes (CTVs) were defined as the entire prostate in the low-risk group, and the entire prostate plus the proximal 1 cm of seminal vesicles in the intermediate-risk group. The CTV to planning target volume (PTV) expansion was 5 mm isometrically in the first and second irradiations. For the third and fourth irradiations, the CTV-to-PTV was expanded 3 to 5 mm isometrically, except for the craniocaudal axis, which was 0 to 2 mm. The prescribed doses for CIRT, described above, ranged from 36 to 44 Gy in a stepwise manner. The dose constraints were as follows: D_1cc_ (rectum) < 100% of the prescribed dose; D_max_ (small intestine) < 50% of the prescribed dose; D_max_ (penile bulb) < 90% of the prescribed dose; and D_mean_ (urethra) < 90% of the prescribed dose. All cases were evaluated daily with bone-matching imaging guidance using orthogonal kilovolt fluoroscopy; however, the insertion of gold seed fiducial markers into the prostate, or perirectal hydrogel spacers, was not mandatory in the present study. To confirm that CIRT was performed according to the treatment plan, a urinary catheter was inserted immediately before treatment, and the dose in the catheter was measured using a linear dosimeter during RT. Urination and defecation were controlled to ensure the reproducibility of the setup.

Low-risk patients did not receive androgen deprivation therapy (ADT), whereas intermediate-risk patients received 4 to 8 months of neoadjuvant ADT. In the present study, however, no detailed restrictions on ADT regimens were imposed.

### Follow-up schedule and statistics

The patients were observed at least once per week during the treatment period. After the completion of treatment, the patients were examined at 1, 3, and 6 months post CIRT, followed by every 3 attend follow-up for up to 3 years post-CIRT. PSA levels and the presence and severity of AEs were confirmed during these visits. Chi-square analysis was used to compare the incidence of AEs, and Welch's *t* test was used compare 2 groups. The Kaplan–Meier method was used to estimate the bRFS, OS, CRFS, and DRFS rates.

## Results

A total of 60 patients who completed the treatment as planned between October 2018 and October 2020 were enrolled in the present study. The median post-CIRT follow-up period was 41.8 months, and the median age at diagnosis was 70 years (IQR, 63-73 years). Of the 60 patients enrolled, 10 were in the low-risk group, whereas 50 were in the intermediate-risk group. All 50 intermediate-risk patients underwent ADT, the median of which was 6.0 months. The median prostate volume before CIRT was 21.1 cm^3^. The median international prostate symptom score (IPSS) was 7, and there were 29 patients with an IPSS score of 8 or more before CIRT. The patient characteristics and treatments are summarized in Table 1.

**Table 1:** Patient characteristics and the treatments

Variable	n = 60
**Median age at diagnosis (IQR), y**	70 (63-73)
**Median iPSA level (IQR), ng/mL**	7.3 (5.6-9.9)
**cT stage**	cT1c: 17 cT2a: 28 cT2b: 3 cT2c: 12
**Gleason score**	3 + 3: 15 3 + 4: 23 4 + 3: 22
**NCCN risk group**	Low: 10 Intermediate: 50
**ADT use**	No: 10 Yes: 50
**Type of ADT**	CAB: 34 LHRH agonist/antagonist alone: 14 Unclear: 2
**Median ADT duration (IQR), mo**	6.0 (5.6-6.6)
**Median prostate volume (IQR), cc**	21.1 (17.7-28.5)
**Median IPSS before CIRT (IQR)**	7 (3-13)
**Diabetes mellitus before CIRT**	No: 56 Yes: 4
**CIRT dose and schedule**	36 Gy/4 fr./1 wk: 10 36 Gy/4 fr./2 wk: 10 40 Gy/4 fr./1 wk: 10 40 Gy/4 fr./2 wk: 10 44 Gy/4 fr./1 wk: 10 44 Gy/4 fr./2 wk: 10

*Abbreviations:* ADT = androgen deprivation therapy; CAB = combined androgen blockade; CIRT = carbon-ion radiation therapy; fr. = fractions; IPSS = international prostate symptom score; LHRH = luteinizing hormone-releasing hormone; NCCN = National Comprehensive Cancer Network; PSA = prostate-specific antigen.

Table 2 shows the incidence of AEs observed in the present study. There was no grade 3 or higher AEs observed in any group, regardless of GU or GI; therefore, no DLTs were observed in the present study. Additionally, no grade 2 or higher GI toxicity was observed in the present study, regardless of the CIRT schedule or dose. The incidence of grade 2 GU toxicity, however, increased with increasing doses, with 10 (50%) of the 20 patients treated with 44 Gy CIRT presenting with grade 2 GI toxicity during the acute phase. Even during the late phase, 5 (25%) patients in the 44 Gy CIRT group experienced grade 2 GU toxicity. The incidence of grade 2 GU toxicity within 90 days of CIRT was significantly higher in the 44 Gy than in the 36 to 40 Gy group (*P* < .01, chi-square test with Yates correction).

**Table 2:** Acute and late toxicities

	Up to 90 days after the start of CIRT CTCAEv4.03		After 91 days from the start of CIRT RTOG/EORTC	
	GU grading	GI grading	GU grading	GI grading
Dose schedule	0 / 1 / 2 / ≥3	0 / 1 / 2 / ≥3	0 / 1 / 2 / ≥3	0 / 1 / 2 / ≥3
36 Gy/4 fr., 1 wk (n = 10)	2 / 7 / 1 / 0	9 / 1 / 0 / 0	7 / 2 / 1 / 0	8 / 2 / 0 / 0
36 Gy/4 fr., 2 wk (n = 10)	4 / 6 / 0 / 0	10 / 0 / 0 / 0	7 / 2 / 1 / 0	9 / 1 / 0 / 0
40 Gy/4 fr., 1 wk (n = 10)	4 / 3 / 3 / 0	10 / 0 / 0 / 0	4 / 5 / 1 / 0	10 / 0 / 0 / 0
40 Gy/4 fr., 2 wk (n = 10)	4 / 5 / 1 / 0	10 / 0 / 0 / 0	8 / 2 / 0 / 0	9 / 1 / 0 / 0
44 Gy/4 fr., 1 wk (n = 10)	3 / 2 / 5 / 0	10 / 0 / 0 / 0	4 / 3 / 3 / 0	9 / 1 / 0 / 0
44 Gy/4 fr., 2 wk (n = 10)	2 / 3 / 5 / 0	9 / 1 / 0 / 0	2 / 6 / 2 / 0	8 / 2 / 0 / 0

*Abbreviations:* CIRT = carbon-ion radiation therapy, CTCAE = Common Terminology Criteria for Adverse Events, F/U = follow-up; fr. = fractions; GI = gastrointestinal; GU = Genitourinary; RTOG/EORTC = Radiation Therapy Oncology Group (RTOG) and the European Organization for Research and Treatment of Cancer.

Next, we analyzed the relationship between prostate volume, baseline IPSS, diabetes mellitus before treatment, and acute or late grade 2 GU toxicity. There were no significant differences in prostate volume between patients who showed grade 2 GU toxicity and those who showed grade 0 to 1 GU toxicity in the acute or late phase (*P* = .946 or *P* = .490). Regarding the baseline IPSS, the median (IQR) IPSS was 10 (7-20) and 6 (3-12) for the 15 patients with acute grade 2 GU and the 45 patients with grade 0 to 1 GU, respectively. Then, the median (IQR) IPSS was 9.5 (5-13) and 7 (3-13) for the 8 patients with late grade 2 GU and the 52 patients with grade 0 to 1 GU, respectively. There were no significant differences in IPSS between patients who showed grade 2 GU toxicity and those who showed grade 0 to 1 GU toxicity in the acute or late phase (*P* = .060 or *P* = .876). Two out of 15 people with acute grade 2 GU and 2 out of 45 people with grade 0 to 1 GU were diabetic, but there was no statistically significant difference between the groups (*P* = .550, chi-square test with Yates correction). Then, none of the 8 patients who presented with late grade 2 GU toxicity had diabetes. Thus, this study found no factors other than dose that affect acute or late grade 2 GU toxicity.

All but 1 patient completed the planned follow-up schedule, as he died of infectious pneumonia 27 months post CIRT. Biochemical failure was observed in 3 patients within the first 3 years post CIRT, although no clinical recurrence or death from prostate cancer was observed (Table 3). The 3-year bRFS, OS, CRFS, and DRFS rates were 95.0%, 98.3%, 100.0%, and 100.0%, respectively. Table 4 shows the clinical backgrounds of the 3 patients who experienced biochemical failure, 2 of which were low-risk (doses of 36 and 40 Gy), whereas the third was intermediate-risk (dose of 40 Gy). Although no clinical signs of recurrence were observed, salvage ADT was initiated in all 3 of these patients, 24 or 27 months post CIRT.

**Table 3:** Clinical outcomes at 3 years after CIRT

	Biochemical failure	Local failure	Distant failure	Patients dead
36 Gy/4 fr., 1 wk (n = 10)	1	0	0	0
36 Gy/4 fr., 2 wk (n = 10)	0	0	0	1*
40 Gy/4 fr., 1 wk (n = 10)	0	0	0	0
40 Gy/4 fr., 2 wk (n = 10)	2	0	0	0
44 Gy/4 fr, 1 wk (n = 10)	0	0	0	0
44 Gy/4 fr., 2 wk (n = 10)	0	0	0	0

*Abbreviation:* fr. = fractions.

⁎ Died of infectious pneumonia.

**Table 4:** List of patients with biochemical failure

	Age at start of CIRT, y	T stage	iPSA, ng/mL	GS	Positive core	CIRT dose/week	ADT	Nadir PSA, ng/mL	Time of recurrence after CIRT
**Case 1**	71	2a	6.10	3 + 3	NR	36 Gy/1 wk	None	2.15	27 mo
**Case 2**	57	2c	6.12	4 + 3	6/14	40 Gy/2 wk	6 mo	<0.01	24 mo
**Case 3**	72	2a	6.06	3 + 3	2/10	40 Gy/2 wk	None	1.89	27 mo

*Abbreviations:* ADT = androgen deprivation therapy; CIRT = carbon-ion radiation therapy; GS = Gleason score; NR = not reported; PSA = prostate-specific antigen.

## Discussion

To the best of the authors’ knowledge, the present study is the first to evaluate and demonstrate the safety and efficacy of 4-fraction CIRT in patients with low- or intermediate-risk localized prostate cancer. No grade 3 or higher AEs, the primary endpoint, were observed in any group, nor were any GI or GU DLTs observed. No grade 2 or higher GI toxicity was observed, regardless of the CIRT schedule or dose. Although the observation period was short, with only 60 cases included, the results of the present study support the low incidence and severity of GI toxicity reported in previous CIRT studies.^13^, ^14^, ^15^, ^16^, ^17^ The 44-Gy group had a significantly higher incidence of acute grade 2 GU toxicity than the 36 to 40-Gy group. No clear association was observed between the treatment schedule (1 vs 2 weeks) and GU toxicity. In addition, this study found no factors other than dose that affect grade 2 GU toxicity. It was previously reported that acute GU toxicity is significantly associated with the development of persistent GU toxicity in patients with prostate cancer undergoing CIRT.^28^ Of note, however, GU toxicity can manifest years after RT,^29^ leading the authors to believe that a CIRT dose of 44 Gy in 4-fractions may be harmful to patients with prostate cancer, regardless of the treatment schedule.

The 3-year bRFS, OS, CRFS, and DRFS rates were 95.0%, 98.3%, 100.0%, and 100.0%, respectively. It would be premature to evaluate outcomes at 3 years post treatment when discussing treatment outcomes for prostate cancer, although, on initial inspection, ultrahypofractionated CIRT seemed to show acceptable antitumor effects in the present study. Considering patients with biochemical failure, a CIRT dose of ≥40 Gy in 4 fractions over the course of 1 week is required in terms of oncologic outcomes. Estimating the biologic equivalent dose from α/β values becomes more difficult as the single dose increases, especially for tumors with low α/β values, such as prostate cancer.^30^ Additionally, the high linear energy transfer of CIRT makes it difficult to determine the biologic equivalent dose using simple calculations.^31^ Tumor control probability (TCP) analysis based on clinical data can solve these difficulties in determining the appropriate dose. The results of TCP analysis in patients with low-risk prostate cancer treated with CIRT were recently reported,^32^ based on which, it was estimated that a CIRT dose of 35.3 Gy in 4 fractions would be required to achieve a 10-year bRFS of 80%.^32^ The results of the present study may, therefore, support the TCP analysis; however, a long-term study with a larger number of patients is essential to validate these findings.

The promising results of the HYPO-RT-PC and PACE-B trials suggest that ultrahypofractionated RT for the treatment of localized prostate cancer will become mainstream in photon beam therapy.^9^^,^^10^ These phase III trials demonstrated that bRFS is not inferior in ultrahypofractionated versus the conventional standard fraction schedule for patients with prostate cancer.^9^^,^^10^ The clinical results of ultrahypofractionated RT with proton beams have also been reported in recent years^33^^,^^34^; however, the superiority of CIRT-based treatment strategies over clinical trials remains premature. The purpose of the present study was to establish a CIRT dose regimen for patients with prostate cancer that could be compared with these other clinical trials. In that sense, the present study is significant. In light of recent studies on perirectal hydrogel spacers, rectal toxicity may become less of a clinical issue in RT for the treatment of prostate cancer in the future.^35^ In addition to bRFS, future studies focusing on appropriate patient selection and GU toxicity are required.

The present study had several limitations. First, this was a single-center study with a small number of patients. A phase II study is currently underway, however, to demonstrate the efficacy of the treatment regimens described herein in a large number of patients (Japan Registry of Clinical Trials ID: 1032220391). The placement of gold seed fiducial markers in the prostate or perirectal hydrogel spacers was not required in the present study. Although GI toxicities in the present study were minor, it is still fair to consider the placement of fiducial markers into the prostate or perirectal hydrogel spacers when comparing the clinical outcomes with other RT modalities. Another limitation of the present study was the lack of information on patient-reported outcomes. Future studies should be comprehensive, and focus not only on physician-reported outcomes but also on patients’ quality of life.

In conclusion, a CIRT dose of 40 Gy in 4 fractions may be effective at balancing the therapeutic effects and toxicity of the therapy. The results of the present study, therefore, support further investigations into the efficacy of this strategy.

## Disclosures

Hitoshi Ishikawa reports financial support was provided by the Ministry of Education, Culture, Sports, Science and Technology of Japan. If there are other authors, they declare that they have no known competing financial interests or personal relationships that could have appeared to influence the work reported in this paper.
